# Awareness, Educational Needs, and Curriculum Preferences Regarding AI and Medical Big Data Education Among Clinical Medicine Undergraduates: Cross-Sectional Survey Study

**DOI:** 10.2196/83441

**Published:** 2026-07-02

**Authors:** Qisha Li, Wenhao Yang, Xiaolan Li, Xiaoqin Li, Ying Li, Ying Cai, Su-Han Jin, Junzhu Xu, Juanyan Shen, Xin Li, Guopin He, Xiaojing Tian, Hu Ma, Jian-Guo Zhou

**Affiliations:** 1 Department of Oncology, The Second Affiliated Hospital of Zunyi Medical University, Intersection of Xinlong Avenue and Xinpu Avenue, Xinpu New District, Zunyi, Guizhou, China, 86 18311543939; 2 School of Management, Zunyi Medical University, Zunyi, China; 3 First Clinical College, Zunyi Medical University, Zunyi, Guizhou, China; 4 Zunyi Medical University, Zunyi, China; 5 The Second Affiliated Hospital of Zunyi Medical University, Zunyi, China; 6 Department of Orthodontics, Affiliated Stomatological Hospital of Zunyi Medical University, Zunyi, Guizhou, China; 7 School of Nursing, Zunyi Medical University, Zunyi, China

**Keywords:** artificial intelligence, AI, medical big data, medical education, undergraduate curriculum, curriculum design

## Abstract

**Background:**

The rapid integration of artificial intelligence (AI) and medical big data into health care is transforming diagnosis, treatment planning, and research. However, formal education in these areas remains limited in undergraduate medical curricula, particularly in China.

**Objective:**

This study aimed to investigate clinical medicine undergraduates’ familiarity with AI and medical big data, their perceived need for related courses, and their preferred curriculum design and assessment methods.

**Methods:**

A cross-sectional, web-based survey was conducted at Zunyi Medical University, Guizhou, China, from January 10 to 17, 2025. In the institutional context of this study, “clinical medicine” included related clinical-track specialties such as pediatrics and psychiatry. All eligible students (N=1094) were invited, and 871 (79.6%) were included in the final analysis. The self-administered questionnaire was developed based on a literature review and expert consultation, with content validity quantified using the content validity index. Descriptive statistics were used to summarize response distributions. For ordinal outcomes (items 1-14), adjusted ordinal logistic regression models were applied, with gender and grade as predictors and major as a covariate. Given the small number of third- and fourth-year students, grade was modeled as an ordered trend variable. For nominal outcomes (items 15-16), group differences were assessed using chi-square tests or Fisher exact tests, as appropriate.

**Results:**

A total of 871 students were analyzed, of whom 62.6% (n=545) were women. Overall familiarity with AI and medical big data was limited: 34.8% (303/871) agreed or strongly agreed that they were familiar with the topic, and only 33% (287/871) reported having at least some prior learning experience. In contrast, the perceived educational need was high: 94% (819/871) considered such a course at least somewhat necessary, 57% (497/871) reported that the course was needed or very needed, 75.5% (658/871) indicated that they would likely or definitely enroll, and 56.5% (492/871) reported that they would likely or definitely engage in self-directed learning. Personalized teaching based on textbooks (566/871, 65%) or open-book examinations (633/871, 72.7%) was the most preferred instructional and assessment format. Preferences for course materials and assessment methods differed by grade but not by gender.

**Conclusions:**

Early-stage clinical medicine undergraduates demonstrated limited familiarity with AI and medical big data but expressed a strong demand for related education. Students preferred structured yet flexible instructional formats and open-book assessments. Although the findings are based predominantly on first- and second-year students, they support the development of staged, practice-oriented AI and medical big data curricula tailored to the needs of early-stage clinical medicine undergraduates.

## Introduction

In recent years, artificial intelligence (AI) technologies such as machine learning, generative AI, and big data analytics have experienced rapid advancement. This evolution is bringing fundamental changes to many fields, especially health care. For example, in clinical diagnostics, deep learning techniques have demonstrated the ability to automatically and accurately detect cancers from histopathological slides and radiological images, potentially reducing repetitive workloads for clinicians [1-4]. AI algorithms also enable intelligent analysis of biosignals such as electrocardiograms and electroencephalograms [5]. Furthermore, AI can enhance clinical decision support systems (CDSSs) by analyzing electronic health records to identify potential diagnostic errors and highlight abnormal results [6-8]. These applications underscore the growing importance of AI in clinical practice and the need to incorporate AI competencies into medical education. Future clinicians must not only master clinical reasoning but also attain AI literacy to effectively apply these tools in patient care. However, the integration of AI-related education into undergraduate medical curricula, particularly in specific regions such as China, appears to lag behind the pace of clinical adoption, leaving many students inadequately prepared to understand and apply these technologies [9-12].

To conceptually inform the design and interpretation of this needs assessment survey, the study drew on selected constructs from the technology acceptance model (TAM) and domains from the Digital Health Competencies in Medical Education (DECODE) framework [13,14]. Rather than formally operationalizing or validating these frameworks, this study used them as organizing perspectives for questionnaire development and interpretation. Specifically, familiarity with AI and medical big data, prior learning experience, and understanding of AI applications were conceptually aligned with perceived ease of use, whereas perceived course necessity, learning intention, and willingness to engage in self-directed learning reflected concepts related to perceived usefulness and behavioral intention within TAM.

In parallel, curriculum-related items concerning learning content, teaching approaches, and assessment preferences were informed by selected competency domains emphasized in the DECODE framework, including data literacy, clinical decision support, and ethical reasoning.

In response to this educational gap, we conducted a cross-sectional survey to assess clinical medicine undergraduates’ familiarity with AI and medical big data, their perceived educational need for related courses, and their preferred instructional and assessment formats. By clarifying current levels of awareness and identifying students’ curriculum preferences, this study aims to provide empirical evidence to inform the design of future AI- and medical big data–related curricula in undergraduate clinical medicine education in China.

## Methods

### Study Design and Participants

This cross-sectional survey study was conducted online among undergraduate students in the clinical medicine category at Zunyi Medical University, Guizhou Province, China. In the institutional context of Zunyi Medical University, this category includes clinical medicine and related clinical-track specialties such as pediatrics and psychiatry. Accordingly, the analyzed sample comprised students from clinical medicine, pediatrics, and psychiatry. The survey primarily targeted first- and second-year students, with a smaller number of third- and fourth-year students also participating.

After providing informed consent, participants completed an anonymous questionnaire distributed via the Wenjuanxing (Changsha Ranxing Information Technology Co Ltd) platform. Participation was voluntary, and respondents were instructed to submit the questionnaire only once. Data collection took place from January 10, 2025, to January 17, 2025.

### Survey Instrument

A self-developed questionnaire was designed based on an extensive review of the literature on AI and medical big data education in clinical medicine and was informed by the TAM and the DECODE framework. The instrument was developed as a needs assessment questionnaire rather than a reflective psychometric scale. Specifically, familiarity with AI and medical big data, prior learning experience, and understanding of AI applications in health care were conceptually aligned with perceived ease of use in TAM, whereas perceived course necessity, enrollment intention, and willingness to engage in self-directed learning were aligned with perceived usefulness and behavioral intention. Curriculum-related items were informed by the DECODE framework, including domains such as data literacy, clinical decision support, and ethical reasoning. Table 1 presents the conceptual mapping between questionnaire domains and selected TAM constructs and DECODE competency domains used to guide questionnaire development and interpretation.

The preliminary questionnaire was reviewed by a panel of 5 experts with experience in medical education, curriculum design, and AI- or medical big data–related research. Experts were selected based on the following criteria: (1) holding at least a Master’s degree and a senior professional title; (2) having at least 3 years of undergraduate teaching or curriculum-related experience; and (3) possessing relevant research or practical experience in medical education, AI, or medical big data.

Content validity was assessed through expert consultation and quantified using the content validity index (CVI). Experts independently rated the relevance and importance of each item using a 5-point scale, with 5 indicating “very important” and 1 indicating “very unimportant.” Ratings of 4 or 5 were considered indicative of item relevance for CVI calculation. The item-level CVI (I-CVI) was calculated as the proportion of experts rating an item as relevant, and the scale-level CVI using the average method was calculated as the average of all I-CVI values. The I-CVI values for the 18 items ranged from 0.80 to 1.00, and the scale-level CVI using the average method was 0.967, indicating good content validity (Multimedia Appendix 1).

**Table 1.** Conceptual mapping of questionnaire domains to selected technology acceptance model (TAM) constructs and Digital Health Competencies in Medical Education (DECODE) competency domains.

Questionnaire domains	Representative questionnaire content	Related TAM construct^a^	Related DECODE competency domain^a^
Awareness and familiarity	Familiarity with AI^b^ and medical big data and understanding of AI applications in health care	Perceived ease of use	Digital literacy
Prior learning experience	Previous exposure to AI and medical big data education	Perceived ease of use	Lifelong learning and digital readiness
Perceived educational need	Necessity and demand for AI and medical big data courses	Perceived usefulness	Professional competency development
Learning intention	Intention to enroll and engage in self-directed learning	Behavioral intention	Self-directed digital learning
Curriculum preferences	Preferences regarding teaching materials and assessment methods	Not directly derived from TAM	Educational implementation and competency-based learning

a The questionnaire was conceptually informed by selected constructs from TAM and domains from the DECODE framework. These frameworks were used as organizing perspectives for questionnaire development and interpretation rather than as formally operationalized or validated theoretical models.

b AI: artificial intelligence.

In addition to quantitative ratings, experts provided qualitative feedback regarding item wording, clarity, comprehensiveness, redundancy, and the appropriateness of response options. On the basis of this feedback, several items were refined to improve readability and conceptual clarity, ambiguous wording was simplified, and response categories were adjusted to enhance consistency and interpretability.

A formal pilot test was not conducted prior to the main survey. However, the questionnaire underwent expert review and refinement to improve item clarity, wording, and comprehensibility before administration.

The questionnaire was developed as an item-based needs assessment instrument rather than a reflective psychometric scale. Although items were conceptually organized into several thematic domains, these domains were intended to describe different aspects of students’ awareness, educational needs, learning intentions, and curriculum preferences rather than to measure unified latent constructs. Accordingly, questionnaire items were analyzed individually, and no total or domain-level summated scores were calculated.

The final questionnaire consisted of 18 items, including 16 single-response items and 2 multiple-response items (Multimedia Appendix 2). The 16 single-response items were used for inferential analyses, whereas the 2 multiple-response items were analyzed descriptively as exploratory findings.

### Data Analysis

Categorical variables were summarized as frequencies and percentages. Because all questionnaire items analyzed in the main inferential analyses were single-item ordinal or nominal responses rather than continuous measures, statistical methods based on normality assumptions were not used. No total questionnaire score or domain-level summated scores were calculated because the questionnaire was designed as an item-based needs assessment instrument rather than a psychometric scale.

For ordinal outcomes (items 1-14), associations with gender and grade were examined using ordinal logistic regression models, with major included as a covariate. Given the small number of third- and fourth-year students, grade was modeled as an ordered trend variable to improve model stability. Odds ratios (ORs) and 95% CIs were reported.

For nominal preference outcomes (items 15-16), group differences were assessed using chi-square tests or Fisher exact tests, as appropriate. The 2 multiple-response items were analyzed descriptively only. The proportional odds assumption was evaluated for ordinal regression models. All analyses were performed in R software (version 4.5.0; R Foundation for Statistical Computing), and a 2-sided *P* value <.05 was considered statistically significant.

### Ethical Considerations

This study was approved by the institutional review board of the Second Affiliated Hospital of Zunyi Medical University (KYLL-2025-057) and conducted in accordance with the Declaration of Helsinki. Informed consent was obtained electronically from all participants prior to data collection. No identifiable personal data were collected; therefore, consent for publication was not required. Each participant received RMB 2 to 5 (RMB 1=US $0.15 as of June 22, 2026) after completing the questionnaire.

## Results

### Participant Characteristics

A total of 871 students were included in the final analysis. Women accounted for 62.6% (545/871) of the sample, and men accounted for 37.4% (326/871). Most respondents were first- or second-year undergraduates (381/871, 43.7% and 457/871, 52.5%, respectively), while only a small proportion were third- or fourth-year students (22/871, 2.5% and 11/871, 1.3%, respectively). In terms of major, 88.7% (773/871) were from clinical medicine, 6.5% (57/871) from psychiatry, and 4.7% (41/871) from pediatrics (Table 2).

**Table 2.** Participant characteristics (N=871).

Characteristics	Participants, n (%)
Gender	
Men	326 (37.4)
Women	545 (62.6)
Grade	
First-year undergraduate	381 (43.7)
Second-year undergraduate	457 (52.5)
Third-year undergraduate	22 (2.5)
Fourth-year undergraduate	11 (1.3)
Major	
Clinical medicine	773 (88.7)
Psychiatry	57 (6.5)
Pediatrics	41 (4.7)

### Awareness and Prior Learning Experience

**Figure 1.**
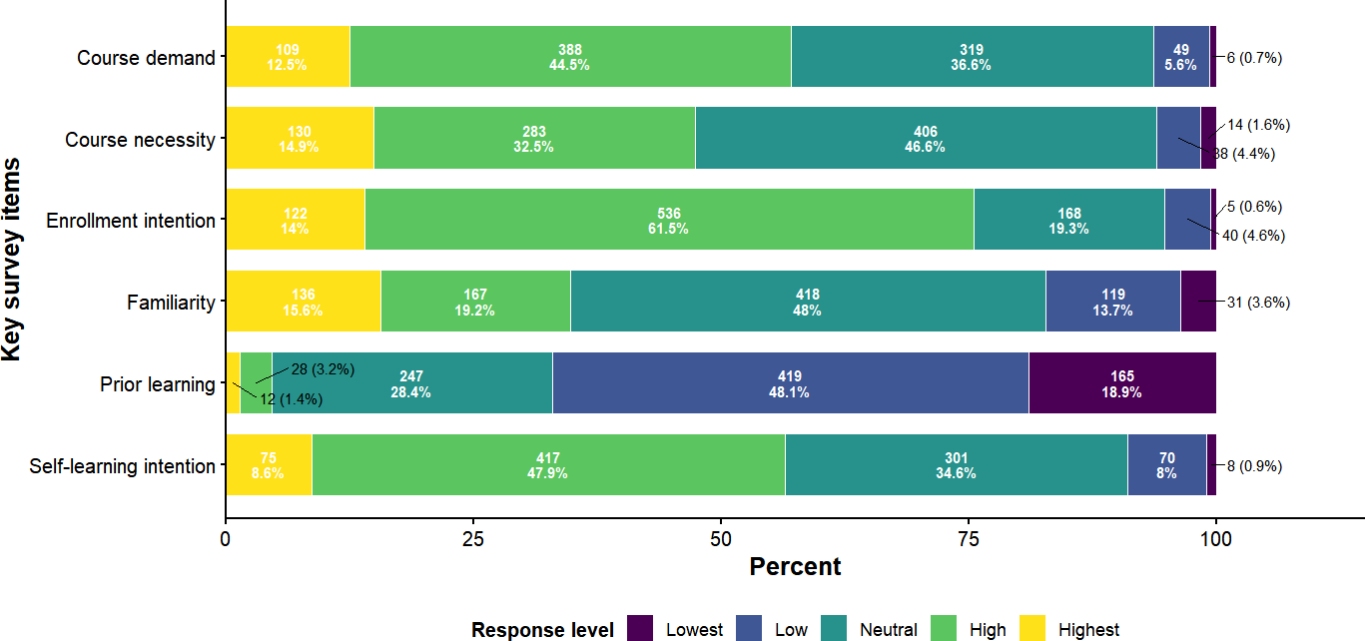
Distribution of responses to 6 key survey items.

Overall, familiarity with AI and medical big data was limited. Only 34.8% (303/871) of respondents agreed or strongly agreed that they were familiar with the topic, whereas 48% (418/871) remained neutral and 17.2% (150/871) disagreed or strongly disagreed. Prior exposure was similarly limited: 33% (287/871) reported at least some learning experience, but only 4.6% (40/871) reported frequent or in-depth learning. A similar pattern was observed for students’ understanding of AI and big data applications in health care (Table 3; Figure 1).

**Table 3.** Overall distribution of key responses regarding artificial intelligence (AI) and medical big data (N=871)^a^.

Domains with response options	Participants, n (%)
Awareness	
Familiarity with AI and medical big data	
Strongly disagree	31 (3.6)
Disagree	119 (13.7)
Neutral	418 (48)
Agree	167 (19.2)
Strongly agree	136 (15.6)
Understanding of AI and big data applications in health care	
Strongly disagree	29 (3.3)
Disagree	130 (14.9)
Neutral	399 (45.8)
Agree	181 (20.8)
Strongly agree	132 (15.2)
Prior learning experience in AI and medical big data	
Never studied	165 (18.9)
Rarely studied	419 (48.1)
Some learning experience	247 (28.4)
Frequent learning	28 (3.2)
In-depth study with practical experience	12 (1.4)
Educational need	
Necessity of offering an AI and medical big data course at the university	
Completely unnecessary	14 (1.6)
Unnecessary	38 (4.4)
Somewhat necessary	406 (46.6)
Necessary	283 (32.5)
Very necessary	130 (14.9)
Demand level for the course	
Completely not needed	6 (0.7)
Not much needed	49 (5.6)
Neutral	319 (36.6)
Needed	388 (44.5)
Very needed	109 (12.5)
Intention to enroll in the course	
Definitely would not enroll	5 (0.6)
Unlikely to enroll	40 (4.6)
Uncertain	168 (19.3)
Likely to enroll	536 (61.5)
Definitely enroll	122 (14)
Intention to self-learn via online resources	
Definitely would not self-learn	8 (0.9)
Unlikely to self-learn	70 (8)
Uncertain	301 (34.6)
Likely to self-learn	417 (47.9)
Definitely self-learn	75 (8.6)
Curriculum preferences	
Preferred type of course materials	
Use fixed textbooks	102 (11.7)
Personalized teaching based on textbooks	566 (65)
Teacher-led flexible teaching	203 (23.3)
Preferred assessment method for the course	
Closed-book examination	152 (17.5)
Open-book examination	633 (72.7)
Paper writing	86 (9.9)

a Percentages may not sum to 100 because of rounding.

### Perceived Need and Learning Intentions

Despite limited familiarity and prior exposure, the perceived educational need was high. Overall, 94% (819/871) of respondents considered it at least somewhat necessary for universities to offer a course on AI and medical big data. In addition, 57% (497/871) reported that the course was needed or very needed, 75.5% (658/871) indicated that they would likely or definitely enroll if such a course were offered, and 56.5% (492/871) reported that they would likely or definitely engage in self-directed learning using online resources (Table 3; Figure 1).

### Curriculum Preferences

**Figure 2.**
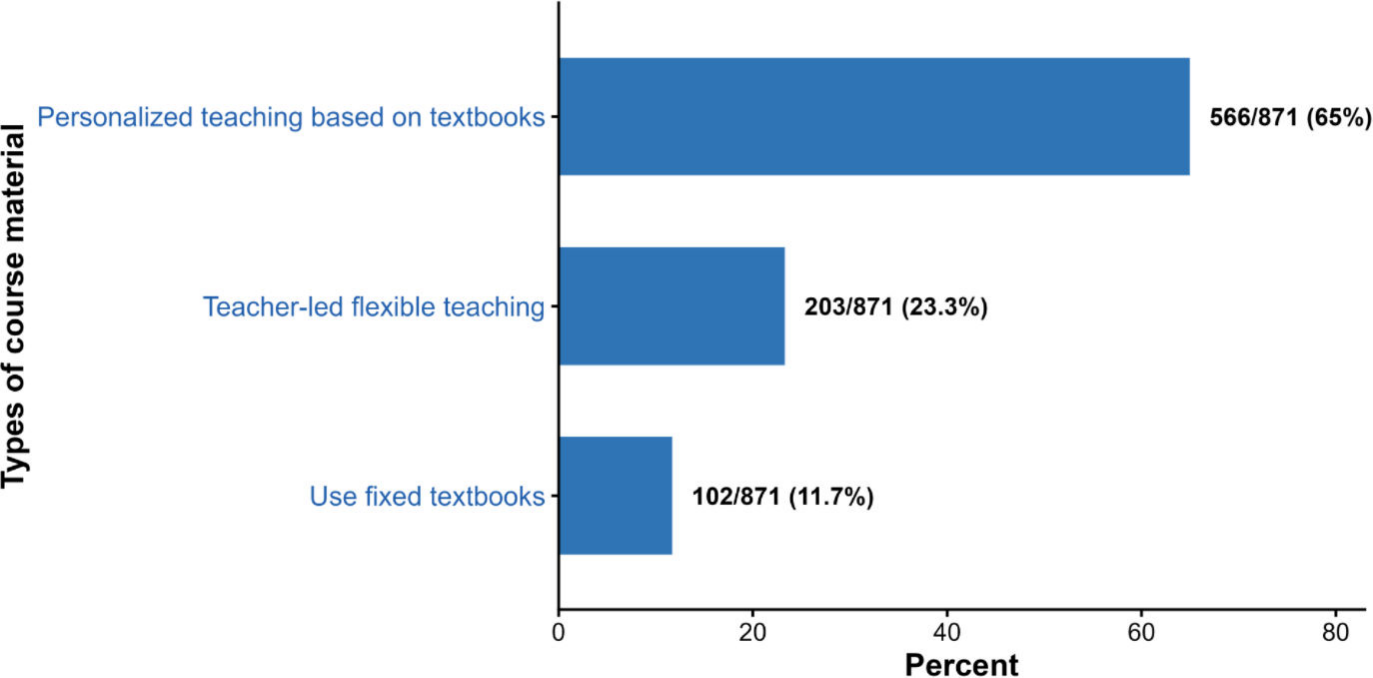
Preferred types of course materials among respondents.

Regarding course design, 65% (566/871) preferred personalized teaching based on textbooks, followed by teacher-led flexible teaching (203/871, 23.3%) and fixed textbooks (102/871, 11.7%). For assessment, open-book examinations were the most preferred format (633/871, 72.7%), followed by closed-book examinations (152/871, 17.5%) and paper writing (86/871, 9.9%; Figures2  3).

**Figure 3.**
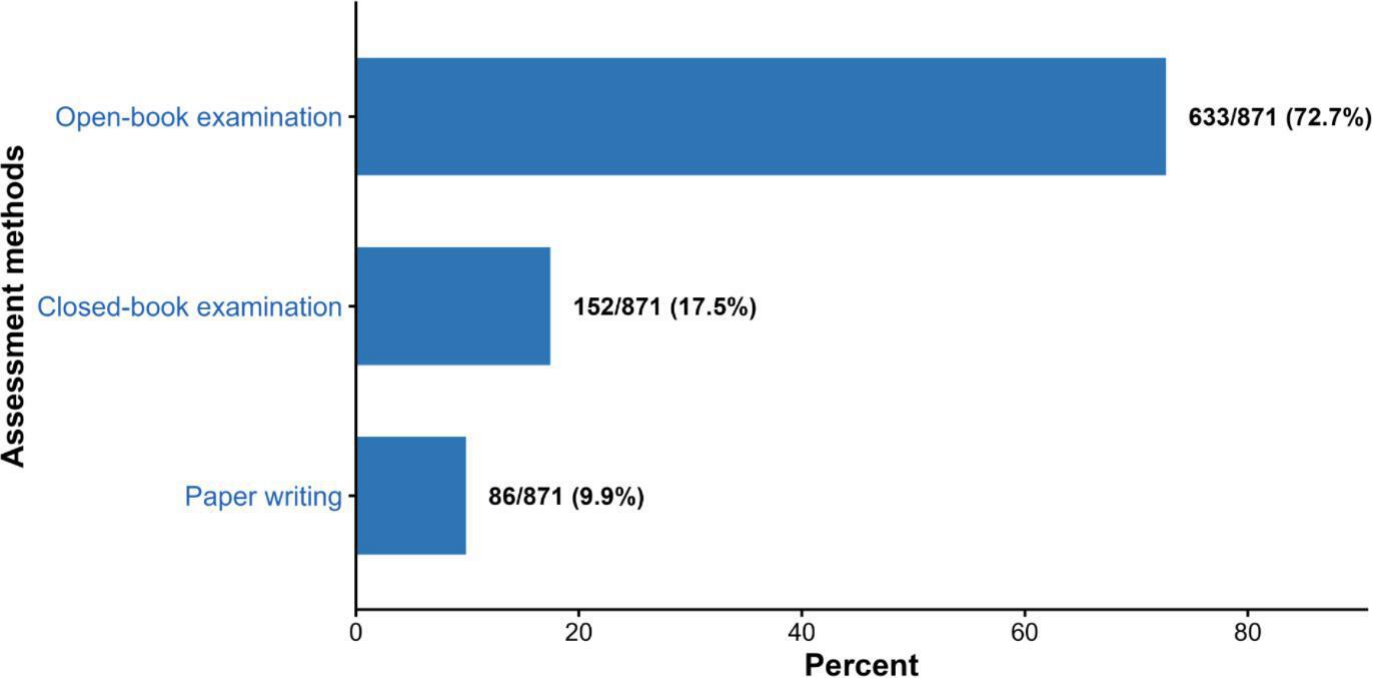
Preferred assessment methods for the proposed course.

### Motivations and Topic Preferences Regarding an AI and Medical Big Data Course

**Figure 4.**
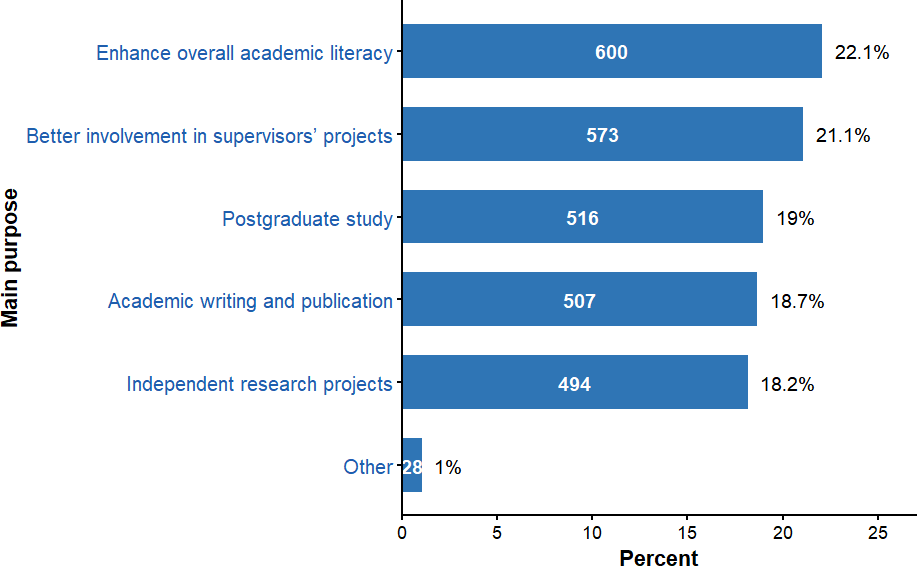
Main purposes for enrolling in the proposed artificial intelligence and medical big data course among clinical medicine undergraduates.

**Figure 5.**
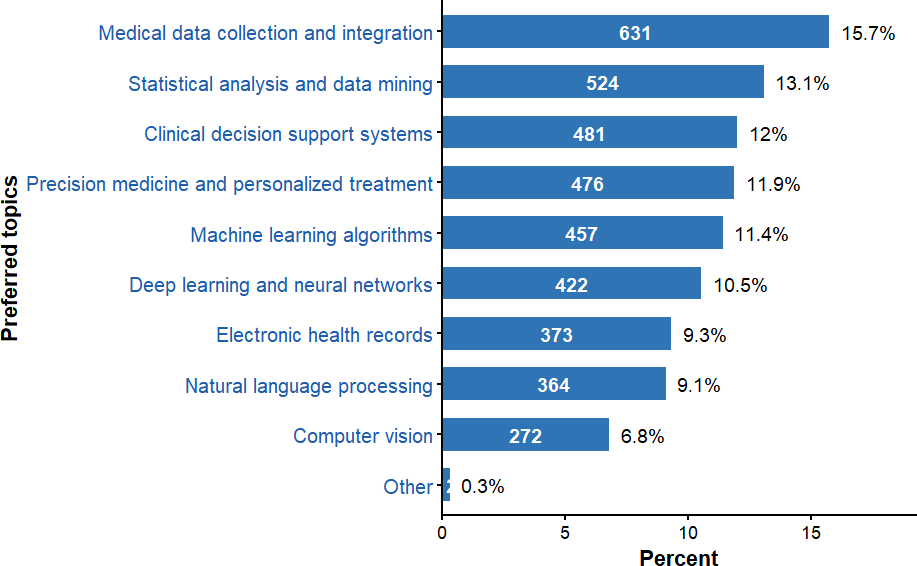
Most needed topics in an “Artificial Intelligence and Big Data in Health Care” course.

Two multiple-response items were analyzed descriptively as exploratory findings. Regarding motivations for taking the course, the most frequently selected purposes were enhancing overall academic literacy (22.1%), better involvement in supervisors’ projects (21.1%), and preparation for postgraduate study (19%; Figure 4). Regarding preferred course content, the most frequently selected topics were data collection and integration (15.7%), statistical analysis and data mining (13.1%), and CDSSs (12%; Figure 5). Because these were multiple-response items, the reported percentages reflect the proportion of total selections rather than the proportion of individual respondents.

### Adjusted Group Differences

In adjusted ordinal logistic regression analyses, female students had lower odds of reporting higher familiarity with AI and medical big data than male students (OR 0.60, 95% CI 0.47-0.78). Increasing grade level was associated with higher familiarity (OR 1.72, 95% CI 1.38-2.14). Similar associations were observed for understanding of AI applications in health care (female vs male: OR 0.54, 95% CI 0.42-0.70; grade: OR 1.77, 95% CI 1.42-2.21) and prior learning experience (female vs male: OR 0.65, 95% CI 0.50-0.85; grade: OR 1.43, 95% CI 1.14-1.79; Table 4).

**Table 4.** Adjusted ordinal logistic regression results for selected outcomes.

Outcomes and predictors	Adjusted odds ratio (95% CI)	*P* value
Familiarity with AI^a^ and medical big data		
Women vs men	0.60 (0.47-0.78)	<.001
Grade (per increase)	1.72 (1.38-2.14)	<.001
Understanding of AI and big data applications in health care		
Women vs men	0.54 (0.42-0.70)	<.001
Grade (per increase)	1.77 (1.42-2.21)	<.001
Prior learning experience in AI and medical big data		
Women vs men	0.65 (0.50-0.85)	.002
Grade (per increase)	1.43 (1.14-1.79)	.002
Necessity of offering an AI and medical big data course at the university		
Grade (per increase)	0.79 (0.63-0.97)	.03
Pediatrics vs clinical medicine	1.95 (1.12-3.40)	.02
Intention to self-learn AI and medical big data via online resources: women vs men	0.72 (0.55-0.94)	.02

a AI: artificial intelligence.

No statistically significant differences were observed by gender, grade, or major for perceived usefulness–related items (items 4-10), overall course demand (item 12), or intention to enroll (item 13). Grade level was associated with lower perceived necessity of offering the course (OR 0.79, 95% CI 0.63-0.97). Pediatrics students had higher odds of reporting greater perceived necessity than clinical medicine students (OR 1.95, 95% CI 1.12-3.40). Female students had lower odds of higher self-reported intention to engage in self-directed learning than male students (OR 0.72, 95% CI 0.55‐0.94).

Preferences for course materials and assessment methods differed by grade level (Fisher exact test: *P*=.007 and *P*<.001, respectively), with no significant differences observed by gender (*P*=.87 and *P*=.14, respectively; Multimedia Appendix 3).

## Discussion

### Principal Findings

This study identified a clear pattern among clinical medicine undergraduates: familiarity with AI and medical big data and prior exposure to these topics were limited, whereas perceived educational need and willingness to learn were high. In other words, students appeared to recognize the importance of AI- and medical big data–related competencies even though many had not yet received systematic training. This pattern is broadly consistent with international surveys from Palestine, Canada, and Germany, as well as recent reviews showing that medical students often report limited formal exposure to AI while simultaneously expressing strong interest in acquiring AI literacy during undergraduate training [10-12,15,16]. From the perspective of the TAM, the observed “low familiarity–high demand” pattern suggests that although students currently report low “perceived ease of use” due to lack of exposure, their “perceived usefulness” of AI for future clinical practice is robust, driving strong behavioral intentions to engage in relevant coursework.

Although familiarity, understanding, and prior learning experience differed by gender and grade, support for course introduction and intention to enroll were broadly consistent across most subgroups. In the adjusted ordinal logistic regression analyses, gender and grade differences were observed in several self-reported measures. Women reported lower levels of familiarity with AI and medical big data, perceived understanding of AI applications in health care, prior learning experience, and self-reported intention to engage in self-directed learning than men. Increasing grade level was associated with higher levels of familiarity, understanding, and prior exposure. These findings should be interpreted cautiously, as all measures were based on self-reported perceptions rather than objective assessments of AI literacy or competence. Therefore, the observed differences reflect variation in perceived exposure and familiarity and should not be interpreted as differences in actual ability or proficiency. No significant gender differences were observed in perceived course necessity or intention to enroll, suggesting broadly consistent support for AI-related education across genders and training stages.

These findings suggest that AI curricula should be designed to be inclusive and supportive, with structured learning opportunities that can enhance confidence and engagement for all students [17,18]. Importantly, the absence of significant subgroup differences in overall course demand and enrollment intention suggests that support for AI-related education may already be widespread, even among students with relatively limited experience. This interpretation is also compatible with earlier work suggesting that students’ willingness to engage with medical AI education may be shaped by perceived usefulness and future relevance rather than by prior technical mastery alone [19].

Students also showed clear preferences regarding curriculum design. Personalized teaching based on textbooks was the most preferred instructional format, and open-book examinations were the most preferred assessment approach. These findings suggest that learners may favor a structured but flexible model of AI education—one that provides conceptual scaffolding through core learning materials while emphasizing understanding, information integration, and application over rote memorization. The exploratory multiple-response items further indicated that students were particularly interested in academic literacy; postgraduate preparation; participation in supervisors’ projects; and course topics such as data collection and integration, statistical analysis and data mining, and CDSSs. These preferences align with recent curriculum-oriented literature emphasizing the progressive integration of AI into existing undergraduate medical education frameworks, authentic learning experiences, and iterative curriculum evaluation rather than isolated or purely technical teaching modules [16,20,21].

Recent competency-oriented literature also provides a useful framework for interpreting our findings. The DECODE international consensus framework for digital health in medical education proposes 4 competency domains, 19 competencies, and a combination of mandatory and discretionary learning outcomes, highlighting that digital health education should extend beyond technical knowledge alone [13]. Similarly, a recent systematic review of physicians’ required competencies in AI-assisted clinical settings emphasized that responsible AI use depends not only on digital and technical skills but also on critical human skills, careful judgment, trust calibration, responsibility, and attention to the patient-physician-AI relationship [22]. Taken together, these frameworks suggest that undergraduate AI curricula should not only be limited to algorithms and tools but should also address appraisal of AI outputs, ethical reasoning, communication, and the safe use of AI in clinical workflows. Given students’ increasing exposure to generative AI tools, this is particularly important for cultivating responsible and clinically appropriate use of such technologies [22,23].

The interpretation of our findings should also consider the Chinese and institutional context. This study was conducted at a single medical university in Guizhou province, and the sample was dominated by first- and second-year students. Therefore, the results should be interpreted primarily as a needs assessment within a single institutional context rather than as a nationally representative estimate of Chinese medical students. Regional variation across China, differences in digital infrastructure, faculty preparedness, and institutional curriculum priorities may all influence how AI education is perceived and implemented. Recent reviews suggest that readiness for AI integration in medical education is shaped by access to digital resources, interdisciplinary collaboration, and implementation support and that institutions with fewer resources may face particular barriers to curriculum adoption [16,21]. Therefore, our findings support local curriculum development while also underscoring the need for future multicenter studies across different regions and medical schools in China.

### Limitations

This study has several limitations. First, this was a single-institution study conducted at 1 medical university in Guizhou province; therefore, the findings may not be fully generalizable to other regions of China with different educational resources, digital infrastructures, or curriculum priorities. Second, the sample was heavily skewed toward first- and second-year students (cumulatively 96.2%), with only a small number of third- and fourth-year students represented. This sampling bias toward early-stage learners limits the external validity of the findings for senior clinical students and constrains the interpretation of grade-related effects. The ordinal trends observed for increasing grade level should be interpreted cautiously, as they are driven predominantly by the contrast between first- and second-year students rather than by robust representation across all training stages. Consequently, the results are best understood as reflecting the perceptions and educational needs of early-stage clinical undergraduates, and future studies should purposively sample students across all years of training to validate these findings. Third, all measures were based on self-reported responses and may be subject to reporting bias. Fourth, although the questionnaire underwent expert consultation and demonstrated good content validity based on CVI results, it was not pilot-tested and was not designed as a reflective multi-item psychometric scale. Therefore, internal consistency reliability testing and exploratory factor analysis were not performed. Future studies should further validate the instrument through pilot testing, test-retest reliability assessment, and psychometric evaluation in broader and more diverse samples. Finally, the cross-sectional design precludes causal inference. Future research should include multicenter and longitudinal studies; examine how AI literacy and curriculum needs evolve across training stages; and evaluate the impact of staged AI curricula on competencies, confidence, and clinical readiness [16,20,21]. Future qualitative or mixed methods research is needed to better understand how gender socialization, prior educational experiences, and institutional factors shape medical students’ AI literacy and confidence.

### Conclusions

This study identified limited familiarity with AI and medical big data but a strong perceived educational need among clinical medicine undergraduates within a single medical university in western China. Students demonstrated consistent interest in structured AI-related education and preferred flexible, application-oriented learning approaches.

These findings support the development of context-specific, competency-oriented AI curricula within the study institution and similar educational settings. However, because the study was conducted at a single institution and included predominantly early-stage students, caution is warranted when generalizing the findings to national medical education contexts.

## Supplementary material

10.2196/83441 Multimedia Appendix 1 Content validity index assessment of the questionnaire based on expert consultation.

10.2196/83441 Multimedia Appendix 2 Supplementary statistical analysis tables, including full adjusted ordinal logistic regression results and group comparisons for course material and assessment preferences.

10.2196/83441 Multimedia Appendix 3 Survey questionnaire.

## Abbreviations

AI: artificial intelligence
CDSS: clinical decision support system
CVI: content validity index
DECODE: Digital Health Competencies in Medical Education
I-CVI: item-level content validity index
OR: odds ratio
TAM: technology acceptance model
