# Supplementary material for: Awareness, Educational Needs, and Curriculum Preferences Regarding AI and Medical Big Data Education Among Clinical Medicine Undergraduates: Cross-Sectional Survey Study
Source: JMIR Form Res. 2026 Jul 2;10:e83441. doi: 10.2196/83441 (PMC13325188; doi:10.2196/83441)
Supplement: Multimedia Appendix 2 [file formative-v10-e83441-s002.docx]

Supplementary Table S1. Full adjusted ordinal logistic regression results for Items 1–14

| Outcome | Predictor | Adjusted OR  (95% CI) | P value |
| --- | --- | --- | --- |
| Familiarity with AI and medical big data | Female vs male | 0.60 (0.47–0.78) | <0.001 |
|  | Grade (per increase) | 1.72 (1.38–2.14) | <0.001 |
|  | Pediatrics vs Clinical Medicine | 1.08 (0.60–1.93) | 0.793 |
|  | Psychiatry vs Clinical Medicine | 0.65 (0.39–1.10) | 0.107 |
| Understanding of AI and big data applications in health care | Female vs male | 0.54 (0.42–0.70) | <0.001 |
|  | Grade (per increase) | 1.77 (1.42–2.21) | <0.001 |
|  | Pediatrics vs Clinical Medicine | 1.24 (0.69–2.23) | 0.471 |
|  | Psychiatry vs Clinical Medicine | 0.69 (0.41–1.14) | 0.145 |
| Prior learning experience in AI and medical big data | Female vs male | 0.65 (0.50–0.85) | 0.002 |
|  | Grade (per increase) | 1.43 (1.14–1.79) | 0.002 |
|  | Pediatrics vs Clinical Medicine | 0.80 (0.43–1.46) | 0.465 |
|  | Psychiatry vs Clinical Medicine | 0.64 (0.37–1.09) | 0.098 |
| Perceived usefulness of AI and medical big data course for career | Female vs male | 1.02 (0.79–1.32) | 0.868 |
|  | Grade (per increase) | 1.02 (0.82–1.26) | 0.890 |
|  | Pediatrics vs Clinical Medicine | 1.08 (0.61–1.92) | 0.799 |
|  | Psychiatry vs Clinical Medicine | 1.11 (0.67–1.84) | 0.696 |
| Perceived impact of AI and medical big data on health care | Female vs male | 1.15 (0.89–1.48) | 0.294 |
|  | Grade (per increase) | 1.04 (0.84–1.28) | 0.736 |
|  | Pediatrics vs Clinical Medicine | 0.69 (0.38–1.25) | 0.221 |
|  | Psychiatry vs Clinical Medicine | 1.14 (0.69–1.88) | 0.616 |
| Research interest enhancement by AI and medical big data course | Female vs male | 0.95 (0.73–1.22) | 0.668 |
|  | Grade (per increase) | 0.88 (0.71–1.09) | 0.245 |
|  | Pediatrics vs Clinical Medicine | 1.04 (0.58–1.84) | 0.903 |
|  | Psychiatry vs Clinical Medicine | 0.79 (0.46–1.35) | 0.381 |
| Helpfulness of the course for understanding research frontiers | Female vs male | 1.18 (0.91–1.53) | 0.221 |
|  | Grade (per increase) | 1.00 (0.81–1.24) | 0.983 |
|  | Pediatrics vs Clinical Medicine | 0.83 (0.46–1.49) | 0.536 |
|  | Psychiatry vs Clinical Medicine | 1.42 (0.84–2.40) | 0.186 |
| Research skills improvement through the course | Female vs male | 1.02 (0.79–1.32) | 0.899 |
|  | Grade (per increase) | 1.00 (0.81–1.24) | 0.976 |
|  | Pediatrics vs Clinical Medicine | 0.93 (0.51–1.69) | 0.805 |
|  | Psychiatry vs Clinical Medicine | 1.20 (0.72–2.00) | 0.494 |
| Research literacy cultivation by the course | Female vs male | 1.13 (0.87–1.47) | 0.360 |
|  | Grade (per increase) | 0.98 (0.79–1.21) | 0.828 |
|  | Pediatrics vs Clinical Medicine | 1.33 (0.75–2.37) | 0.326 |
|  | Psychiatry vs Clinical Medicine | 1.32 (0.78–2.23) | 0.297 |
| Value of AI and medical big data tools for the future | Female vs male | 1.16 (0.90–1.50) | 0.253 |
|  | Grade (per increase) | 1.11 (0.90–1.38) | 0.332 |
|  | Pediatrics vs Clinical Medicine | 0.91 (0.52–1.60) | 0.747 |
|  | Psychiatry vs Clinical Medicine | 1.59 (0.95–2.67) | 0.079 |
| Necessity of offering an AI and medical big data course at university | Female vs male | 0.98 (0.75–1.27) | 0.851 |
|  | Grade (per increase) | 0.79 (0.63–0.97) | 0.028 |
|  | Pediatrics vs Clinical Medicine | 1.95 (1.12–3.40) | 0.018 |
|  | Psychiatry vs Clinical Medicine | 1.00 (0.59–1.68) | 0.996 |
| Demand level for the course | Female vs male | 1.06 (0.81–1.37) | 0.675 |
|  | Grade (per increase) | 0.93 (0.75–1.16) | 0.515 |
|  | Pediatrics vs Clinical Medicine | 1.03 (0.58–1.82) | 0.932 |
|  | Psychiatry vs Clinical Medicine | 0.79 (0.47–1.34) | 0.380 |
| Intention to enroll in the course | Female vs male | 0.86 (0.65–1.14) | 0.297 |
|  | Grade (per increase) | 0.92 (0.73–1.15) | 0.452 |
|  | Pediatrics vs Clinical Medicine | 0.96 (0.51–1.80) | 0.895 |
|  | Psychiatry vs Clinical Medicine | 0.87 (0.49–1.53) | 0.620 |
| Intention to self-learn AI and medical big data via online resources | Female vs male | 0.72 (0.55–0.94) | 0.016 |
|  | Grade (per increase) | 1.03 (0.83–1.28) | 0.790 |
|  | Pediatrics vs Clinical Medicine | 1.20 (0.67–2.16) | 0.535 |
|  | Psychiatry vs Clinical Medicine | 0.73 (0.43–1.24) | 0.245 |

Note: Ordinal logistic regression models were adjusted for gender, grade, and major. Grade was modeled as an ordered trend variable. Odds ratios greater than 1 indicate higher odds of reporting a higher response level, whereas odds ratios less than 1 indicate lower odds of reporting a higher response level.

Supplementary Table S2. Associations of course material and assessment preferences with gender and grade

| Outcome | Comparison | Statistical test | P value |
| --- | --- | --- | --- |
| Preferred type of course materials | Gender | Pearson’s chi-square test | 0.871 |
| Preferred type of course materials | Grade | Fisher’s exact test | 0.007 |
| Preferred assessment method for the course | Gender | Pearson’s chi-square test | 0.136 |
| Preferred assessment method for the course | Grade | Fisher’s exact test | <0.001 |

Note: Fisher’s exact test was used for comparisons by grade because of sparse cell counts in higher-year groups.
